# Evaluation of Novel Preformed Particle Gel System for Conformance Control in Mature Oil Reservoirs

**DOI:** 10.3390/gels10010070

**Published:** 2024-01-17

**Authors:** Abdulaziz Almakimi, Ahmed Ben Ali, Ibnelwaleed A. Hussein, Baojun Bai

**Affiliations:** 1 Petroleum Engineering Department, Missouri University of Science and Technology, Rolla, MO 65409, USA; aaay35@mst.edu; 2 Gas Processing Center, Qatar University, Doha P.O. Box 2713, Qatar; ahmed.benali@qu.edu.qa (A.B.A.); ihussein@qu.edu.qa (I.A.H.)

**Keywords:** carboxymethyl cellulose, conformance control, excessive water production, plugging efficiency, preformed particle gel

## Abstract

To address challenges associated with excessive water production in mature oil reservoirs, this study introduces a carboxymethyl cellulose (CMC)-based material as a novel preformed particle gel (PPG) designed to plug excessive water pathways and redistribute the subsequent injected water toward unswept zones. Through microwave-assisted grafting copolymerization of CMC with acrylamide (AM), we successfully generated multi-sized dry particles within the range of 250–800 µm. Comprehensive analyses, including Fourier-transform infrared spectroscopy (FTIR) and scanning electron microscopy (SEM), have confirmed the chemical composition and morphology of the resulting carboxymethyl cellulose-grafted crosslinked polyacrylamide (CMC/PAMBA). Swelling kinetics and rheology tests were conducted to confirm the ability of this novel PPG system to perform at different reservoir conditions. The results of core flooding experiments showed that the CMC/PAMBA PPG is capable of plugging open fractures with a water breakthrough pressure gradient of up to 144 psi/ft. This preformed particle gel (PPG) system was designed specifically for application in Middle East reservoirs, which are distinguished by high salinity and elevated temperature levels. This PPG system is able to swell up to 10 times its original size in seawater and maintain a strength of about 1300 Pa at a temperature of 80 °C. Further optimization is conceivable to enhance injection efficiency and achieve superior plugging outcomes.

## 1. Introduction

With the world’s increasing energy demands, the application of Improved Oil Recovery (IOR) techniques becomes crucial for tapping into mature reservoirs that hold significant quantities of untapped oil. As conventional reservoirs deplete, their natural production rates decline, necessitating advanced methods to enhance oil extraction efficiency. IOR involves the implementation of various technologies, such as water flooding, gas injection, or chemical treatments, to optimize oil recovery from existing wells. Mature oil reservoirs that have undergone extended periods of water flooding and are characterized by declining production rates and increased water cuts present a unique challenge in the field of petroleum engineering. As these reservoirs age, the need for efficient methods to enhance oil recovery and reduce excessive water production becomes increasingly crucial. During water flooding operations, the injected water or gas tends to flow through high-permeability zones, like fractures or channels, leaving a substantial amount of oil untapped. This results in poor sweep efficiency or a conformance issue [1]. The need to treat the significant volume of produced water will add a substantial load on the facilities, consequently diminishing the feasibility of the producing well. 

Various methods can be employed to address conformance problems, including the use of viscosity-enhancing materials and plugging agents. Gel applications for conformance control have emerged as a promising strategy to block high-permeability zones and divert the injected fluids toward low-permeability zones [2]. A successful application of gel treatment in a heterogeneous reservoir will result in a reduction in water production and an increase in oil production, as a result of sweep efficiency modification. Two major types of gel treatments were applied: in situ gels and preformed particle gels. In the context of in situ gels, a low-viscosity gelant is injected into the reservoir. Once the gelling conditions are met, this gelant transforms into a bulk gel, serving to block the targeted zone [3]. However, in situ gels have their drawbacks, including chromatographic separation during injection and uncertainties surrounding gelation conditions. Furthermore, they exhibit limited plugging efficiency in large fractures and may cause damage to the adjacent oil-bearing matrix. Preformed particle gel (PPG) was introduced in 1996 to address the limitations of in situ gel systems. PPGs are pre-crosslinked gel particles that offer size and strength controllability. PPGs have the ability to swell up to 200 times their original size when mixed with water before injection [4]. They have proven to be effective in blocking fractures and fracture-like features without causing damage to the oil-bearing formation [5]. Preformed particle gels are applied successfully in more than 10,000 wells in China and the United States [6].

Many proposed and commercially available preformed gels are comprised of synthetic polymer-based polyacrylamide derivatives materials [7,8,9,10,11], crosslinked with organic agents such as N,N′-methylene bisacrylamide (MBA) [12], polyethylene glycol diacrylate (PEGd) [13], divinylbenzene (DVB) [14], 1,6-hexanediol diacrylate [15], or inorganic crosslinkers like chromium [16], potassium [17], and aluminum [18]. Additionally, various monomers like styrene sulfonate [19] and dimethyldiallylammonium chloride [20], as well as additives like clay, silica, and graphene [21,22], are introduced into the formulation to enhance swelling, elevate resistance to high temperatures, and modulate strength. 

Polysaccharides, distinguished by their exceptional properties such as biodegradability, renewability, nontoxicity, and cost-effectiveness, offer a promising avenue for sustainable PPG development [23,24,25,26]. Among these, carboxymethyl cellulose (CMC), a modified cellulose, emerges as a noteworthy candidate with versatile applications across diverse industries including food, pharmaceuticals, water treatment, drilling muds, and many other fields [27,28,29,30]. In the realm of materials science, the utilization of polysaccharides in various applications has been hindered by their inherent limitations compared to synthetic polymers. To overcome these constraints, chemical modifications have been strategically employed to enhance properties such as heat and abrasion resistance, mechanical strength, water or oil repellency, thermal stability, and antibacterial activity [31,32,33,34]. An effective approach to impart novel attributes to these natural polymers involves the grafting of synthetic polymer chains onto their molecular backbones. The advancement of polysaccharide-based grafted materials has been a focal point of extensive research endeavors over the years [24,35,36,37]. These materials, harnessed through three primary pathways known as the “grafting through” process, “grafting onto” process, and “grafting from” process, have demonstrated versatility in diverse applications. Their utility extends across various applications [38,39,40,41,42,43,44,45,46]. 

Despite the manifold applications of polysaccharide-based materials, only a limited number of studies have proposed PPGs constructed from these natural polymers. Notably, works by Baloochestanzadeh et al. [47] and Zhang et al. [48] utilized starch-grafted polyacrylamide loaded with nanosilica and alginate/polyacrylamide, respectively. Elaf et al. [49,50] introduced PPGs based on chitosan. This study aims not to supplant existing polyacrylamide-based PPGs but to mitigate their environmental impact by incorporating CMC into their structure. Additionally, this research seeks to endow the prepared PPGs with enhanced characteristics. Addressing key parameters highlighted by Esfahlan et al. [51], such as particle size, swelling capacity in saline solutions, swollen particle strength, and thermal stability, the focus is on developing multi-sized PPGs based on natural polymers. These PPGs are envisaged to exhibit a high swelling ratio in high-saline media, augmented mechanical strength, and enhanced thermal stability at elevated temperatures, contributing to the advancement of environmentally sustainable and performance-optimized PPGs.

In this paper, a systematic approach was followed to evaluate the performance of the newly developed CMC/PAMBA PPG under various conditions of salinity, pH, and temperature. Core flooding experiments were conducted to assess the plugging efficiency of this novel PPG system. 

## 2. Results and Discussion

### 2.1. Chemical Structure

**Figure 1:**
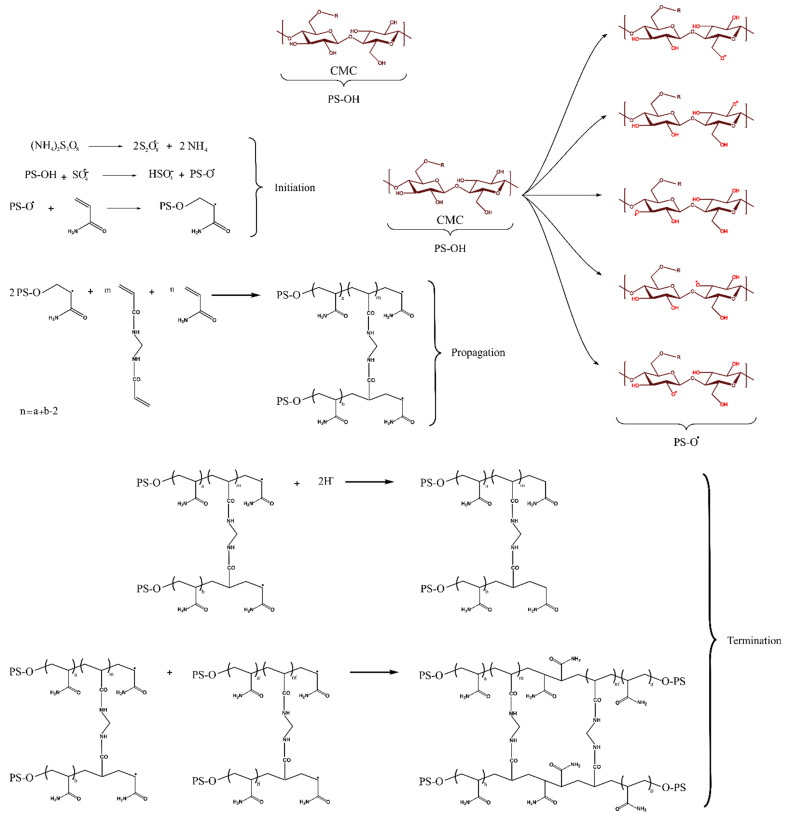
Proposed grafting copolymerization mechanism for the synthesis of CMC/PAMBA.

The grafting copolymerization of AM onto the CMC backbone is initiated by APS. Upon decomposition, APS generates sulfate radicals, which abstract hydrogen atoms from the hydroxyl groups of CMC, generating radicals along the CMC chains. These radicals serve as initiators for the grafting copolymerization by attacking the double bonds in AM. The resulting AM radicals initiate a chain reaction as they react with other AM molecules. Concurrently, AM molecules can also engage in reactions with MBA, a crosslinker containing two vinyl groups susceptible to radical attacks. This leads to the formation of crosslinked structures between the grafted chains, enhancing the structural integrity of the material. The grafting copolymerization reaction can be terminated through various mechanisms, including the recombination of two copolymer radicals, effectively concluding the chain growth process, as explained in Figure 1.

**Figure 2:**
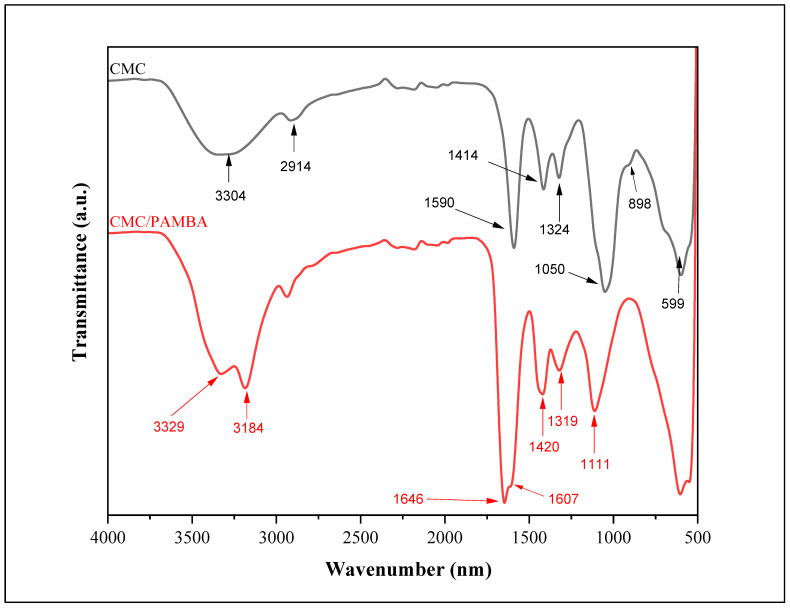
FTIR spectra of CMC and CMC/PAMBA.

**Figure 3:**
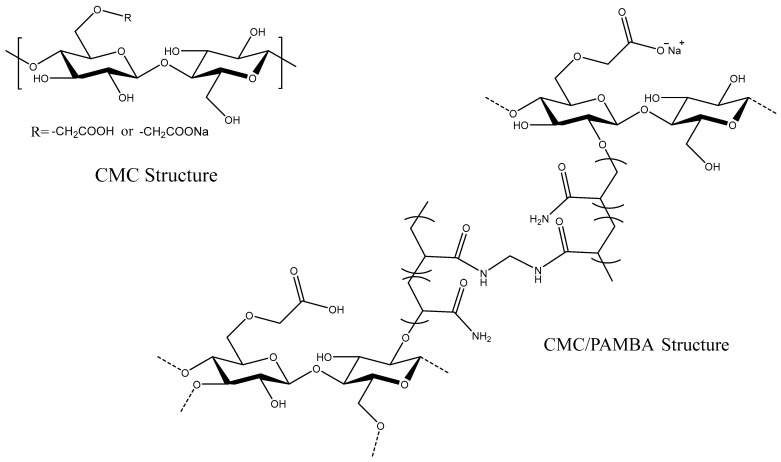
Chemical structure of CMC and CMC/PAMBA.

The confirmation of successful CMC/PAMBA synthesis and its chemical structure was achieved by comparing its FTIR spectrum with that of CMC (Figure 2). In the FTIR spectrum of CMC, a broad band in the 3700–3000 cm^−1^ region signified the stretching vibration of the OH group. The peak at 2914 cm^−1^ was attributed to a C–H stretching vibration. Strong absorption bands at 1590 cm^−1^ and 1414 cm^−1^ corresponded to the asymmetric and symmetric stretching modes of the carboxylate anion, while the peak around 1324 cm^−1^ resulted from an O–H bending vibration. The characteristic C–O vibrations from alcohol were observed at 1050 cm^−1^, and β1–4 glycoside bonds between glucose units were evident at 898 cm^−1^. In the FTIR spectrum of CMC/PAMBA, the absence of the C=C strong stretching peak between 995 and 900 cm^−1^ indicated the successful conversion of AM and MBA. The grafting onto the CMC backbone was confirmed by new peaks related to both PAM and MBA. Bands at 3184 cm^−1^ and 3329 cm^−1^ were assigned to the symmetric and asymmetric stretching of the N–H bond. Vibration bands at 1646 cm^−1^ and 1607 cm^−1^ were attributed to C=O stretching and N-H bending, while peaks at 1420 cm^−1^ and 1319 cm^−1^ were related to C–H bending and C–N stretching, respectively. The shifting peak from 1050 cm^−1^ to 1100 cm^−1^ in CMC/PAMBA compared to CMC, typically associated with a strong C–O stretch, could be explained by the appearance of C–O–C groups resulting from the grafting reaction of hydroxyl functions with the π-bond of PAM and MBA. The proposed chemical structure of the prepared PPG, as depicted in Figure 3, aligns with findings from previous studies [52,53].

### 2.2. Morphology

**Figure 4:**
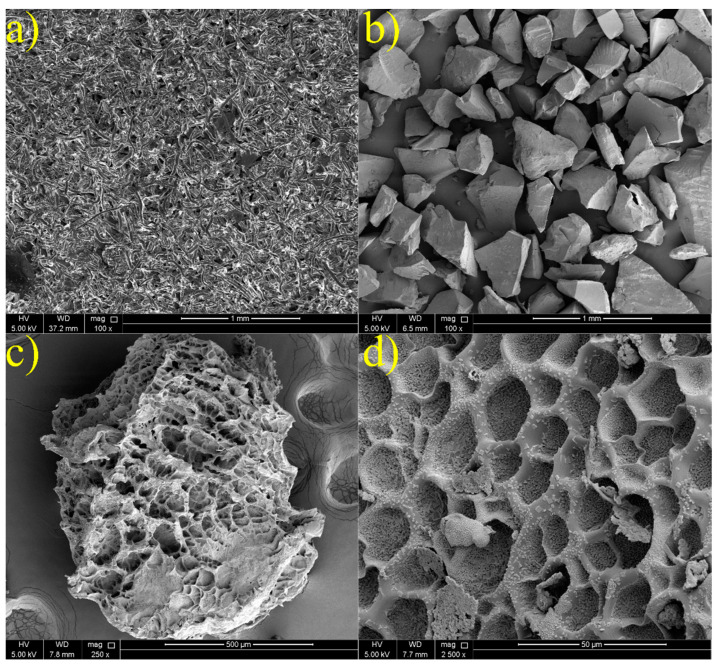
SEM images of (**a**) CMC, (**b**) dry CMC/PAMBA, and (**c**,**d**) swollen CMC/PAMBA in brine.

Figure 4 illustrates the surface morphologies and microstructures of both CMC and CMC/PAMBA in both the dried state and after swelling. The PPG exhibits an appearance of irregular angular granules with rough surfaces and microporous structures, in contrast to the fibrillar surface of CMC, as observed in various studies [53,54,55]. Notably, the dried state of the PPG shows an absence of visible pores. Pores become evident on the particle surface only upon swelling, and these pores play a crucial role in the absorbency of the PPG. Different pore shapes are observable, encompassing a wide range of sizes within the limit of 10 to 70 µm.

### 2.3. Swelling Behaviour and Rheology

The effects of salinity, pH, and temperature on the swelling and rheological properties of the CMC/PAMBA PPG were assessed. Four brine samples were prepared: distilled water, 2% KCl, 10-times diluted seawater (10 DSW), and seawater (SW). The seawater used in this study was Persian Gulf seawater. To study the effect of pH on the swelling kinetics and gel rheology, five samples were prepared. The influence of temperature on the CMC/PAMBA PPG samples, prepared in distilled water, was evaluated over a temperature range from room temperature (23 °C) to 80 °C.

#### 2.3.1. Effect of Salinity

**Figure 5:**
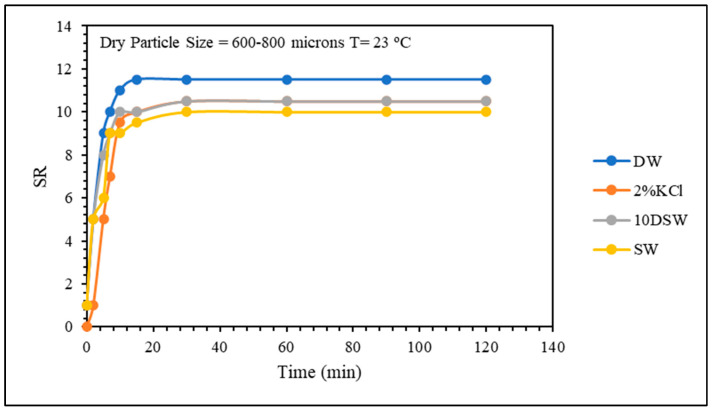
Salinity effect on swelling kinetics.

**Figure 6:**
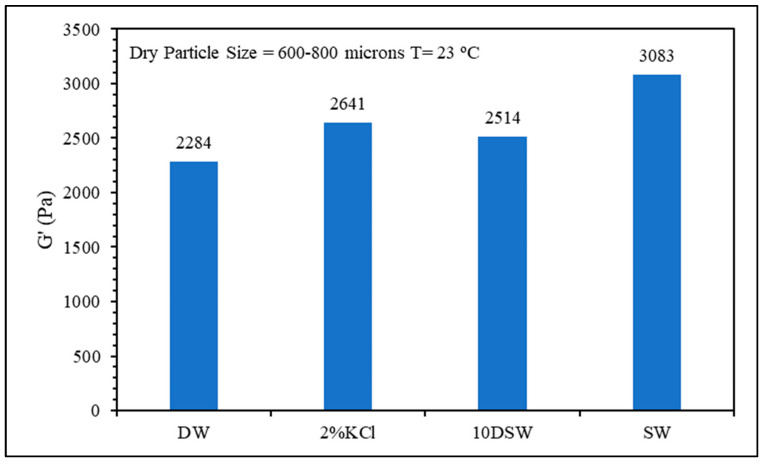
Salinity effect on gel strength.

The effect of water salinity on the swelling kinetics and PPG strength is shown in Figure 5 and Figure 6. The gel sample prepared in distilled water is the fastest to reach the equilibrium swelling ratio, which is also the highest. Conversely, the gel prepared in seawater has the lowest equilibrium swelling ratio. For the first 10 min, the gel sample prepared in distilled water has the fastest swelling rate, while the other samples have close swelling rate and ratio values. This phenomenon is attributed to the concentrated effect of Na^+^, Ca^2+^, and Mg^2+^ in brine solutions. The presence of these ions reduces the osmotic pressure difference, which serves as the driving force for swelling between the PPG and the solution. Additionally, those cations can engage in ionic interactions with the -COO^−^ groups present in the PPG structure. This interaction results in a shielding effect, where the cations replace the polymer–water interaction with a polymer–salt interaction. Consequently, this alteration diminishes the swelling ratio of the material. The combined influence of the osmotic pressure and shielding effect leads to a substantial reduction in water uptake by CMC/PAMBA when exposed to brine solutions, highlighting the intricate interplay between the material structure and the surrounding salt environment.

Rheology measurements were conducted to assess gel strength. Strength is a deterministic factor for the plugging efficiency of the gel, as weak gels are more prone to deformation under lower pressure gradients, potentially creating channels for post-water flow. Gels prepared with lower salt content displayed lower storage moduli compared to gel prepared in seawater. In general, increasing the swelling will reduce the gel strength. 

#### 2.3.2. Effect of pH

**Figure 7:**
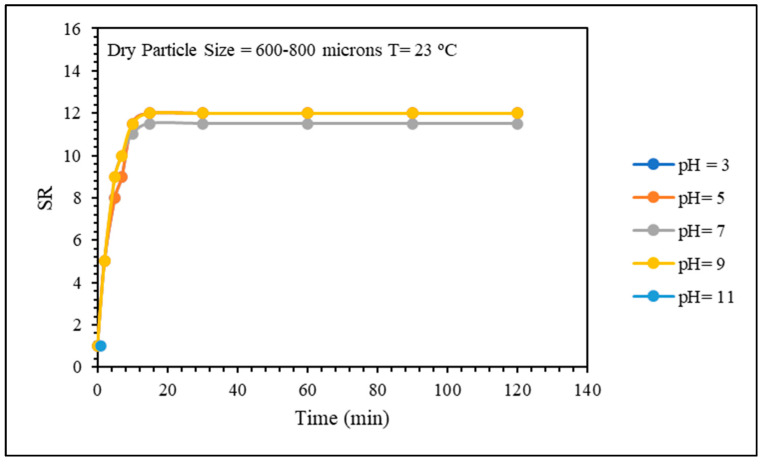
pH effect on swelling kinetics.

**Figure 8:**
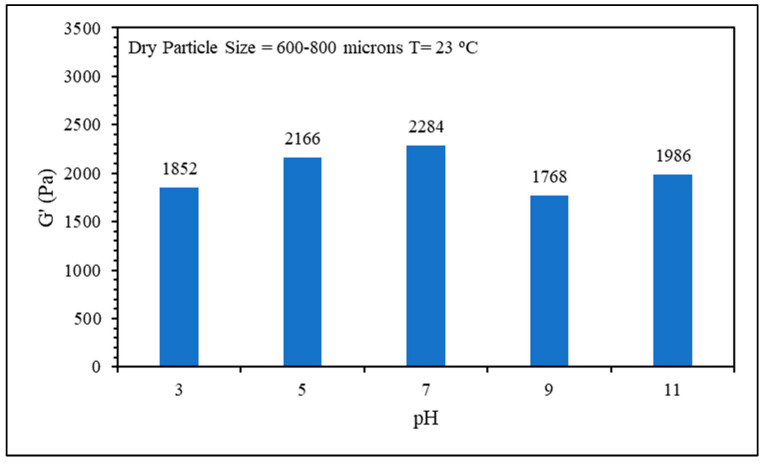
pH effect on gel strength.

Five solutions with different pH values ranging from 3 to 11 were prepared to evaluate the effect of pH on the swelling kinetics of the CMC/PAMBA PPG. The effect of pH on the swelling kinetics was almost negligible, and the swelling ratios of all five solutions were very close during the measurement (Figure 7). The storage modulus values were also close, as shown in Figure 8. It is well known that the carboxylic groups in the material are affected by the pH, both functional groups COOH and COO^−^ coexist simultaneously in the CMC/PAMBA structure, and their relative proportions vary depending on the pH of the solution. At pH = pKa (with pKa being 4.3 for CMC), the quantities of COOH and COO^−^ are equal. Below pH ≤ pKa, carboxyl groups (-COOH) predominate, while above pH ≥ pKa, carboxylate groups (COO^−^) become more prominent. In the latter state, the chemical structure of the grafted material is expected to be more expanded due to the repulsion force between anionic charges along the material. This expansion would theoretically lead to an enhancement in the swelling ratio and a parallel decrease in the strength. However, our experimental observations deviate from this expectation. Despite the anticipated repulsion forces and increased availability of carboxylate groups at higher pH, the swelling ratio did not exhibit the anticipated enhancement.

#### 2.3.3. Effect of Temperature

**Figure 9:**
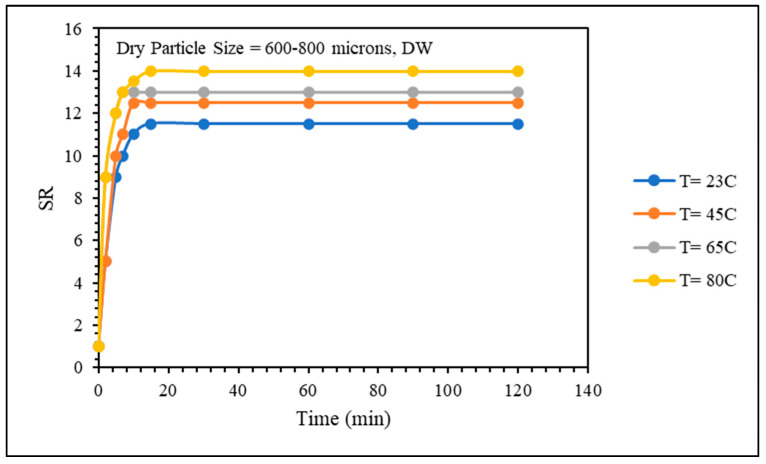
Temperature effect on swelling kinetics.

**Figure 10:**
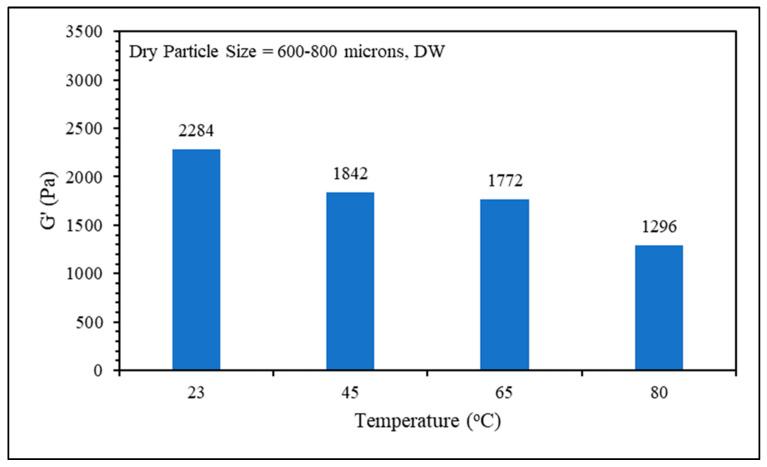
Temperature effect on gel strength.

The swelling kinetics and rheological properties of the gel samples were evaluated over a temperature range from room temperature (23 °C) to 80 °C. As depicted in Figure 9, it is evident that higher temperatures accelerate the swelling kinetics of the gel and increase the equilibrium swelling ratio. Additionally, Figure 10 illustrates that the gel sample at the lowest temperature exhibits greater strength compared to the gel sample prepared at lower temperatures. As the temperature rises, the PPG absorbs heat, causing structural expansion and enhanced water uptake. This phenomenon, elucidated by Ben Ali et al. [10], results in material weakening. Bai et al. [5] proposed another explanation: at temperatures above 60 °C, hydrolysis of amino groups (–CONH2) to carboxylate groups (–COO–) occurs, contributing to increased swelling and water uptake in PPG-based polyacrylamide. This dual mechanism highlights the intricate interplay between temperature, chemical transformations, and the resulting material properties.

### 2.4. Core Flooding Test

**Figure 11:**
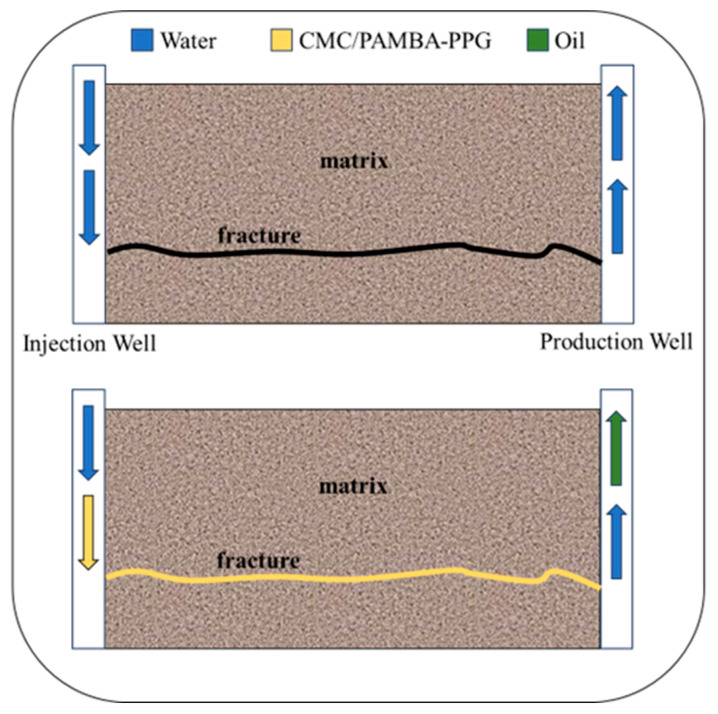
Before gel placement, injected water flows through the fracture, causing excessive water production and low oil recovery due to conductivity differences. Gel filling the fracture redistributes water, reducing water production and increasing oil recovery.

To assess the injectivity and plugging efficiency of the CMC/PAMBA PPG in fracture models, five experiments were conducted. The impact of gel particle size and injection flow rate was investigated in core flooding tests. In these experiments, initial water injection at a flow rate of 1 mL/min commenced until pressure stabilization. Subsequently, the swelled gel was injected at the designated flow rate until the pressure stabilized, and the average stabilized pressure was recorded. To determine plugging efficiency, post-water floods were conducted at flow rates of 0.1, 0.25, 0.5, 0.75, and 1 mL/min. At each flow rate, the pressure was allowed to stabilize to calculate the residual resistance factor (Frr). In Figure 11, the effect of the plugging efficiency is shown when the gel particles fill the fracture and improve the sweep efficiency.

#### 2.4.1. Effect of Particle Size

Three gel samples were prepared to evaluate the effect of particle size on the gel injection and plugging efficiency: the base case of dry particle sizes of 600–800 microns, the small particle size of 250–600 microns, and a mix of equal ratios between the two sizes. 

**Figure 12:**
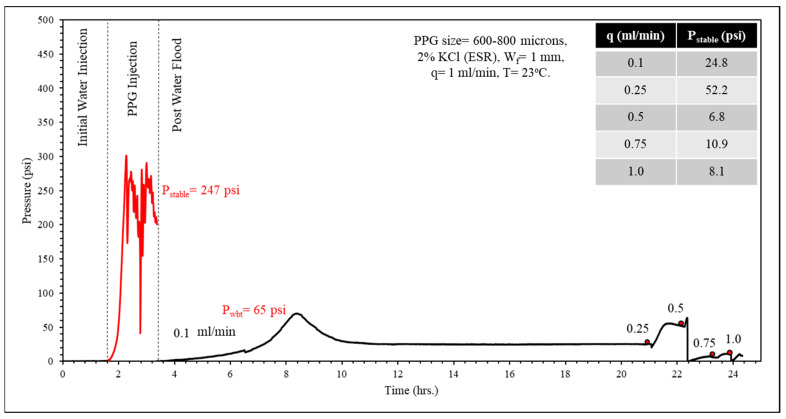
Core flooding data for PPG (dry size 600–800 microns) fully swollen in 2% KCl in a fractured sandstone core (Experiment #2).

In Figure 12, the gel particles were initially dry with a size of 600–800 microns and fully swelled in 2% KCl brine. They were injected at a flow rate of 1 mL/min into a fractured sandstone core with a fracture width of 1 mm. The average stabilized pressure during gel injection was approximately 247 psi. The post-water flood initiated at a flow rate of 0.1 mL/min, with the water breakthrough pressure at about 65 psi, stabilizing at approximately 25 psi. The flow rate was incrementally increased, and at a flow rate of 0.5 mL/min, the gel began to be produced in the effluent for a short period, causing the pressure to decrease and stabilize at about 7 psi. The same procedure was repeated for other particle sizes. 

**Figure 13:**
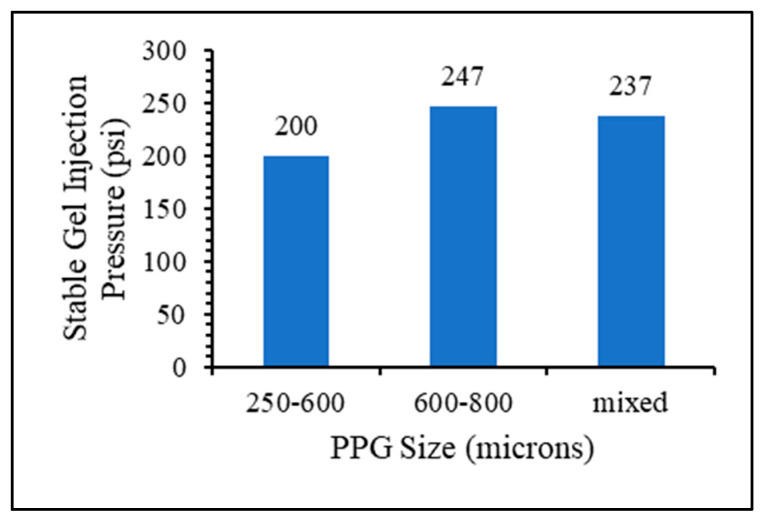
Gel particle size effect on gel injection pressure.

The stable gel injection pressure was recorded for three experiments (Figure 13), indicating that a smaller particle size corresponded to a lower recorded injection pressure. However, in the experiment involving a mix of particle sizes used in equal amounts, the stable injection pressure fell between that of the larger and smaller particles.

**Figure 14:**
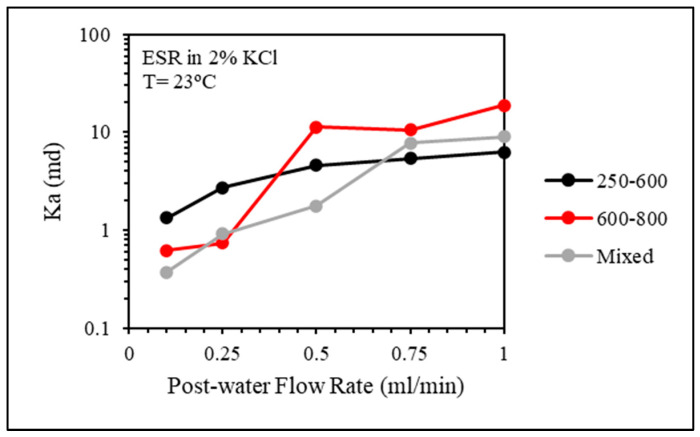
CMC/PAMBA PPG particle size effect on the permeability of the fractured core after gel placement.

**Figure 15:**
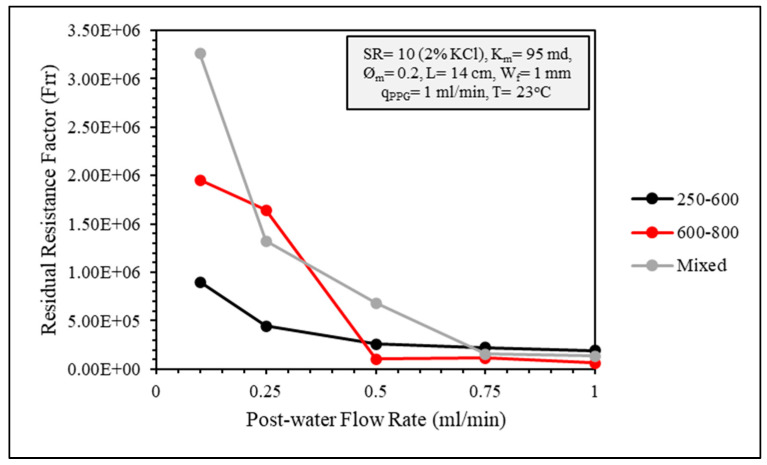
Residual resistance factor (Frr) of different particle sizes.

To evaluate the plugging efficiency of the CMC/PAMBA PPG in fractured sandstone cores, the permeability of the fractured core was calculated along with the residual resistance factor. In Figure 14, it is evident that the smaller particle size exhibited better packing within the fracture, as the permeability values showed no significant changes with increasing post-water injection flow rates. In the other two experiments involving larger particle sizes, although the permeability was initially lower during the post-water flooding, it increased with higher flow rates. This observation suggests that larger particles were more prone to deterioration, potentially creating channels. In Figure 15, the residual resistance factor was calculated to represent the plugging efficiency of the gel, and the high values of Frr suggested a plugging efficiency as high as 99%. 

#### 2.4.2. Effect of Gel Injection Flow Rate

**Figure 16:**
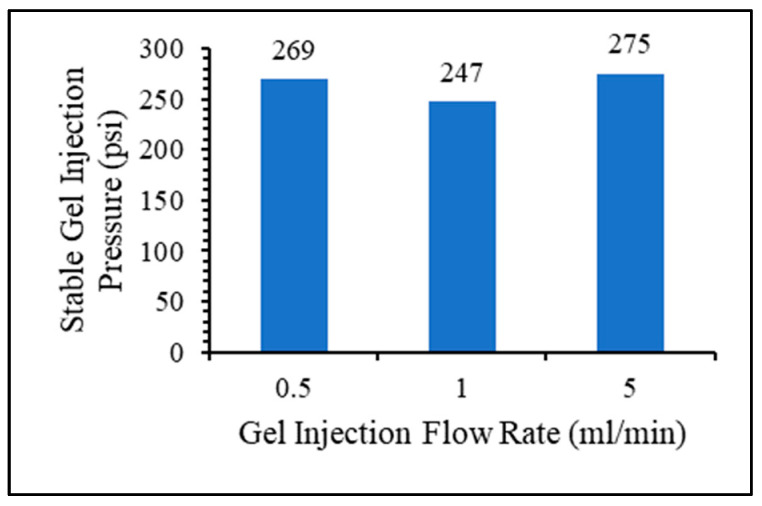
Gel injection flow rate effect on gel injection pressure.

The effect of gel injection flow rate on the injection pressure and plugging efficiency was investigated using three flow rates: 0.5, 1, and 5 mL/min. In Figure 16, the effect of the gel injection flow rate on the stable injection pressure seems to be minor. 

**Figure 17:**
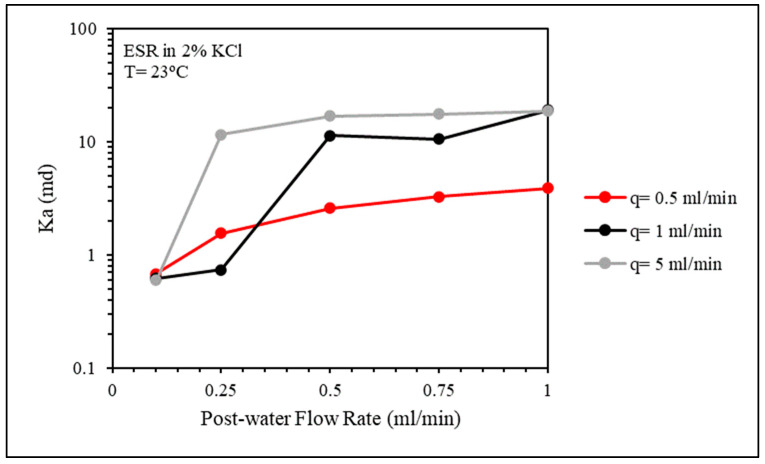
CMC/PAMBA PPG injection flow rate effect on the permeability of the fractured core after gel placement.

**Figure 18:**
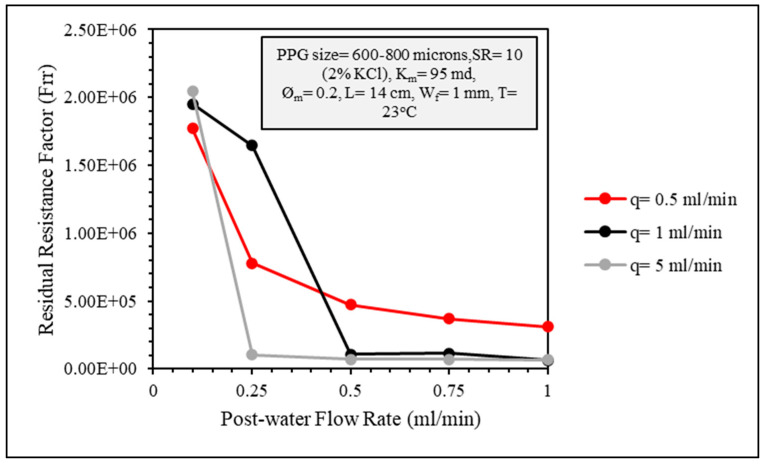
Residual resistance factor (Frr) of different gel injection flow rates.

The permeability of the fractured core after gel placement was at close values when the post-water flow rate was 0.1 mL/min. However, as the water flow rate increased, the lowest gel placement flow rate of 0.5 mL/min showed more durability (Figure 17). The Frr values were also high, indicating very good plugging efficiency (Figure 18).

#### 2.4.3. Gel Dehydration

Gel dehydration refers to the loss of water from the gel particles due to the pressure difference between the fracture and the matrix, leading to the gel being concentrated. Gel dehydration can significantly affect the gel’s propagation into the fracture. As the gel becomes more concentrated, it also becomes stronger, requiring a higher pressure gradient for proper placement. Furthermore, severe gel dehydration can deteriorate the gel pack coherency and result in low residual resistance factors at low water injection flow rates. 

**Figure 19:**
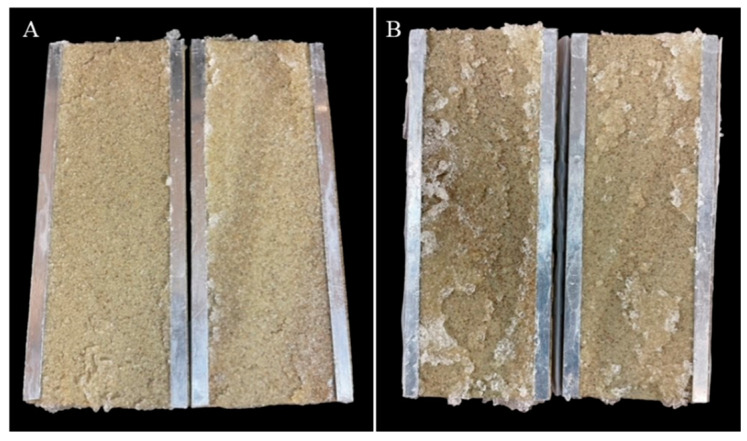
CMC/PAMBA PPG after post-water flood; smaller particles (**A**) showed better packing than larger particles (**B**).

A quick assessment of CMC/PAMBA PPG dehydration was conducted by monitoring the effluent particle size during gel injection for both large (600–800 microns) and small (250–600 microns) particles using the weight difference method. In this method, the collected (dehydrated) gel particles are weighed and then placed in an oven at a temperature of 80 °C for 3 days until completely dry, with no change in weight noticed. The difference between the water weight absorbed during the initial swelling and the water weight evaporated during the drying process represents the amount of water lost during the gel injection. Dividing this by the initially absorbed water weight and multiplying by 100 gives the dehydration percentage. The small particles dehydrated by approximately 9%, while the large particles dehydrated by about 17%. Figure 19 displays the gel after post-water flood for both sizes, and the minor dehydration observed for the smaller particles resulted in better packing.

## 3. Conclusions

We introduced a novel polysaccharide-based material as a PPG, namely carboxymethyl cellulose-grafted crosslinked polyacrylamide (CMC/PAMBA). This material was synthesized through a microwave-assisted grafting copolymerization process, utilizing APS as the initiator and MBA as the crosslinker. The comprehensive characterization of the grafted material employed various techniques including Fourier-transform infrared spectroscopy (FTIR), scanning electron microscopy (SEM), rheology, swelling measurements, and core flooding tests in an open fracture model. The key conclusions drawn from our investigations are as follows:

FTIR analysis provided evidence of successful grafting reactions occurring between the hydroxyl groups of CMC and the vinyl groups of both AM and MBA.

SEM images corroborated the grafting copolymerization process, revealing the formation of micropores responsible for the material’s absorbency.

The equilibrium swelling ratio of the CMC/PAMBA PPG shows less sensitivity to salinity and pH value, while higher temperatures result in faster swelling.

Core flooding experiments demonstrate the effective plugging efficiency of the CMC/PAMBA PPG in open fractured models, with a minor impact of gel injection flow rate on stable pressure. 

Small particle sizes of CMC/PAMBA PPG exhibited better packing and less dehydration, resulting in stable plugging. 

Future developments suggested for this study include improving the plugging efficiency performance at higher post-water injection rates by adding a self-healing property to the carboxymethyl cellulose-grafted crosslinker polyacrylamide PPG, as this property will help in forming a strong, coherent bulk gel within the fracture to resist higher pressure gradients.

## 4. Materials and Methods

### 4.1. Sources

The biopolymer used in this study, carboxymethyl cellulose (CMC, ≥99%, DS: 0.86), was purchased from Arshine Food Additives Co., Ltd., Changsha, China. Acrylamide (AM, 98%), N,N′-methylenebisacrylamide (MBA, 99%), and ammonium persulfate (APS, ≥99%) were obtained from Glentham Life Sciences Ltd., Corsham, UK. All the chemicals were used as received without any further purification. All the solutions were prepared in deionized (DI) water.

### 4.2. Material

A blend comprising 0.2 g of ammonium persulfate (APS) initiator and 1 g of carboxymethyl cellulose (CMC) was dissolved in 60 mL of deionized water and subjected to a 2 min reaction period under stirring and irradiation at 600 W power within a microwave reactor. Subsequently, 4 g of acrylamide (AM) was dissolved in 15 mL of water, followed by the addition of a specified quantity of N,N′-methylenebisacrylamide (MBA) as a crosslinker. The grafting reaction was initiated by introducing the AM/MBA solution into the reaction mixture, with continuous irradiation until gel formation occurred. The grafted material was then recovered from the reaction vessel through crushing and subsequently purified using deionized water to eliminate unreacted reagents or non-crosslinked material. Finally, the preformed gel CMC/PAMBA was dried in an oven at 65 °C until a constant weight was achieved and was subsequently ground with a mortar, yielding a powder with varying particle sizes ranging from 250 to 800 µm after sieving. Table 1 below shows the name, quantity, and chemical structure of the material used for the synthesis procedure.

Cores: Brea Sandstone cores (2-inch diameter) were used to build the fracture model. A water-saturated cylindrical core of known porosity and permeability was cut lengthwise equally into two halves, stainless-steel strips were fixed on the core face using epoxy, then the core was wrapped with Teflon. The stainless-steel strips’ thickness represents the fracture width. 

Brine: A 2% KCl solution was prepared and used as a base case for gel plugging efficiency evaluation. Persian Gulf Sea water was prepared for swelling kinetics evaluation. The recipe can be found in (Hamza et al., 2019) [56].

### 4.3. Methodology

Chemical structure: Fourier-transform infrared spectroscopy (FTIR) using Perkin Elmer Spectrum was recorded for the CMC and CMC/PAMBA in the wave number range of 500–4000 cm^−1^ to confirm the grafting copolymerization and formation of PPGs. 

Morphology: The morphological structures of both dried and swollen CMC/PAMBA in brine were visualized using scanning electron microscopy (SEM). Prior to SEM analysis, the PPG underwent three freeze-drying stages for 48 h using the Labconco FreeZone 12 machine. The freeze-drying stages comprised (1) placing the PPG samples in the freeze-dryer chamber, chilling to −50 °C at a rate of 0.6 °C/min, and maintaining an isothermal plateau for 2 h; (2) subjecting the samples to a sublimation stage at −15 °C under a total gas pressure of 18 Pa; and (3) drying the samples at a higher shelf temperature of 25 °C under a pressure of 10 Pa to achieve optimal residual moisture content. Subsequently, SEM analysis was conducted using an FEI Nova Nano SEM 450 microscope. ImageJ software (1.54) was employed for measuring the pore sizes.

Swelling behavior: Understanding the swelling behavior of the gel is essential for field applications to optimize the injection mode and design the proper particle size for plugging the targeted zone. The swelling kinetics of the CMC/PAMBA PPG were evaluated by mixing a dry PPG sample of known volume in different brine samples and recording the change in volume with time. Typically, 1 mL of dry PPG is immersed in a 50 mL tube of brine, shaken, and left to swell. The swelling ratio (SR) was calculated using Equation (1) [51].

(1) $$SR=\frac{V_{Swelled}}{V_{dry}}$$

**Figure 20:**
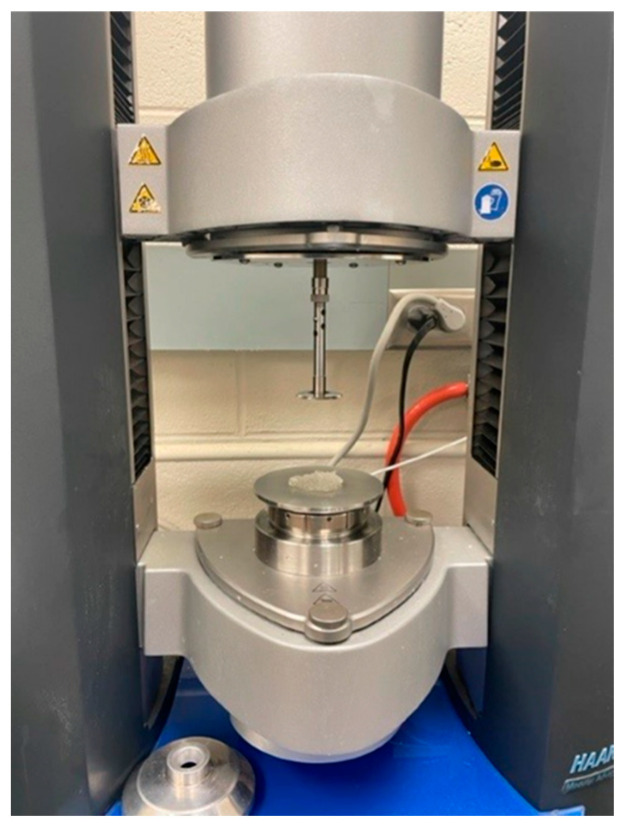
Haake MARS III rheometer used to measure the elastic modulus of CMC/PAMBA PPG samples.

Rheology evaluation: The elastic modulus (G′), often referred to as Young’s modulus, is a measure of a material’s stiffness or rigidity. In the context of preformed particle gels (PPGs), the elastic modulus represents the gel’s ability to deform under applied stress and return to its original shape when the stress is removed. The higher the elastic modulus, the higher the gel strength. Maintaining optimal mechanical integrity, coupled with practically acceptable injectivity, is crucial for gel applications aimed at reducing excessive water production. The elastic modulus (G′) in Pascals (Pa) of swollen CMC/PAMBA PPG samples was acquired through dynamic oscillatory measurements using a Haake MARS III rheometer (Figure 20). To ascertain the linear viscoelastic region of the gel sample, strain sweep experiments were conducted. The measurements were performed at a 1% strain value with a 1 mm gap height between the sensor and the plate containing the PPG samples. The frequency was set to 1.00 Hz [57,58].

**Figure 21:**
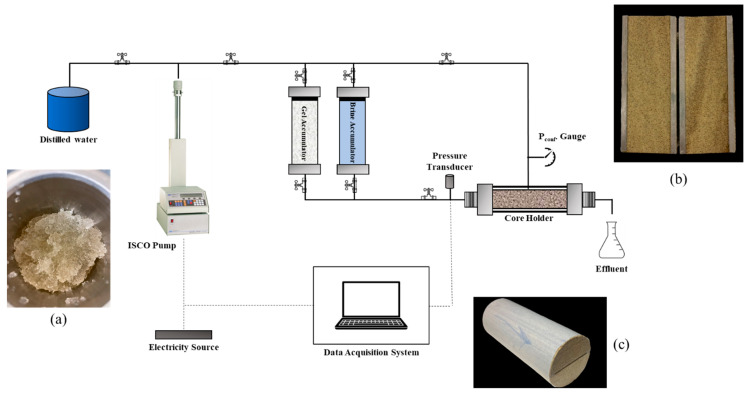
Core flooding setup. (**a**) is the swollen gel packed in the accumulator, (**b**) is the fracture model, and (**c**) is the wrapped fracture model inserted in the core holder.

Core flooding test: To evaluate the plugging efficiency of the CMC/PAMBA PPG, core flooding experiments were conducted using fractured sandstone cores. A core flooding setup was assembled (Figure 21), consisting of an ISCO pump, two accumulators for the gel sample and brine, and a Hassler-type core holder. The sandstone cores were placed in the oven at a temperature of 120 °C to ensure residual water was removed before they were used. They were then vacuumed for 24 h in a vacuum cell. A 2% KCl brine was used to saturate the cores for another 24 h. Porosity was calculated using the water saturation method, and initial brine permeability was calculated using Darcy’s law. 

To create the fracture model, the fully saturated sandstone core was divided into two equal parts using an electric saw. Two stainless-steel strips of the intended fracture thickness were attached to the interior of the separated core sections. The prepared fractured sandstone core was then wrapped with Teflon and inserted into the core holder. Following the securement of the connection lines, a confining pressure exceeding the injection pressure by a minimum of 500 psi was applied. Initial brine injection was conducted at 1 mL/min until the pressure stabilized. PPG dry particles were swollen in 2% KCl brine, placed in the accumulator, and injected at the designated flow rate. After the pressure stabilized, the injection process was terminated, and lines were cleaned and prepared for post-water flood. Post-water flooding was conducted at flow rates of 0.1, 0.25, 0.5, 0.75, and 1.0 mL/min, until the pressure stabilized for each flow rate. The residual resistance factor (Frr) is a common term used to assess the plugging efficiency of the injected PPG, and it is calculated using the following equation [57]:

(2) $$F_{rr}=\frac{K_{b}}{K_{a}}$$

where:

K_b_ is the initial permeability of the fractured sandstone model including the matrix and fracture.
K_a_ is the permeability of the fractured sandstone model after the CMC/PAMBA PPG placement.

**Figure 22:**
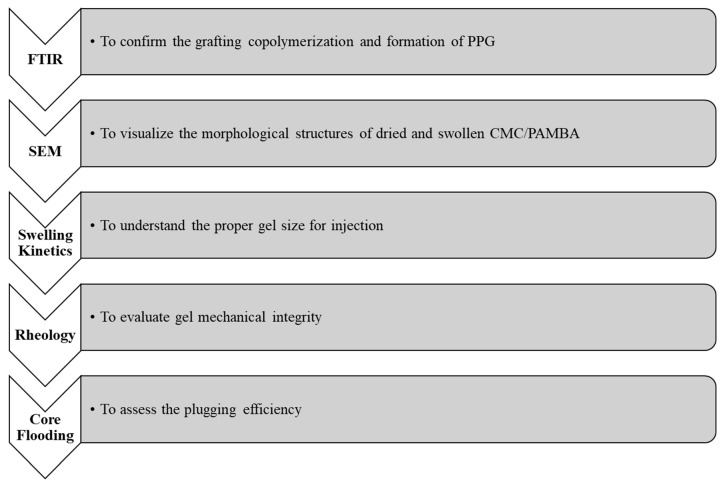
Experimental procedure.

Five core flooding experiments were conducted to investigate the effects of gel particle size and injection flow rate on the gel injection pressure and the plugging efficiency. All experiments were performed at room temperature. Table 2 below shows the core flooding experiments. The experimental procedure is illustrated in Figure 22.

## Figures and Tables

**Table 1:** Materials used for the synthesis of CMC/PAMBA.

Name	Chemical Structure	Amount (g)
Carboxymethyl cellulose (CMC)	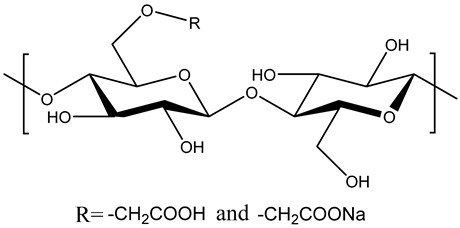	1
Acrylamide (AM)	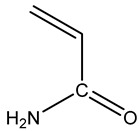	4
N,N′-Methylenebisacrylamide (MBA)	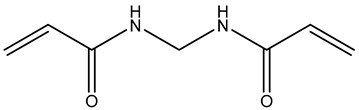	
Ammonium Persulfate (APS)	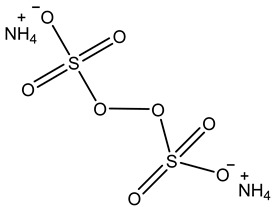	0.2

**Table 2:** Core flooding experiments.

Exp. #	Core Matrix Permeability (md)	Core Matrix Porosity	Core Length (cm)	Fracture Width (mm)	Gel Dry Particle Size (microns)	Brine	SR	Gel Injection Flow Rate (mL/min)	Temp. (°C)
1	95	0.2	14	1.0	600–800	2% KCl	10	0.5	23
2								1.0	
3								5.0	
4					250–600			1.0	
5					Mixed			1.0	

## Data Availability

All data and materials are available on request from the corresponding author. The data are not publicly available due to ongoing research using part of the data.
